# Improved Bone Quality and Bone Healing of Dystrophic Mice by Parabiosis

**DOI:** 10.3390/metabo11040247

**Published:** 2021-04-16

**Authors:** Hongshuai Li, Aiping Lu, Xueqin Gao, Ying Tang, Sudheer Ravuri, Bing Wang, Johnny Huard

**Affiliations:** 1 Department of Orthopaedic Surgery, University of Pittsburgh, Pittsburgh, PA 15260, USA; yit3@pitt.edu (Y.T.); bingwang@pitt.edu (B.W.); 2 Steadman Philippon Research Institute, Vail, CO 81657, USA; alu@sprivail.org (A.L.); xgao@sprivail.org (X.G.); sravuri@sprivail.org (S.R.)

**Keywords:** Duchenne muscular dystrophy, bone abnormality/healing, parabiosis, circulating factors and progenitors

## Abstract

Duchenne muscular dystrophy (DMD) is a degenerative muscle disorder characterized by a lack of dystrophin expression in the sarcolemma of muscle fibers. DMD patients acquire bone abnormalities including osteopenia, fragility fractures, and scoliosis indicating a deficiency in skeletal homeostasis. The dKO (dystrophin/Utrophin double knockout) is a more severe mouse model of DMD than the mdx mouse (dystrophin deficient), and display numerous clinically-relevant manifestations, including a spectrum of degenerative changes outside skeletal muscle including bone, articular cartilage, and intervertebral discs. To examine the influence of systemic factors on the bone abnormalities and healing in DMD, parabiotic pairing between dKO mice and mdx mice was established. Notably, heterochronic parabiosis with young mdx mice significantly increased bone mass and improved bone micro-structure in old dKO-hetero mice, which showed progressive bone deterioration. Furthermore, heterochronic parabiosis with WT C56/10J mice significantly improved tibia bone defect healing in dKO-homo mice. These results suggest that systemic blood-borne factor(s) and/or progenitors from WT and young mdx mice can influence the bone deficiencies in dKO mice. Understanding these circulating factors or progenitor cells that are responsible to alleviate the bone abnormalities in dKO mice after heterochronic parabiosis might be useful for the management of poor bone health in DMD.

## 1. Introduction

Duchenne muscular dystrophy (DMD) is the most common of the muscular dystrophies and is caused by mutations in the dystrophin gene [1,2]. Progressive weakness and degeneration of muscle tissue due to the lack of dystrophin usually results in the loss of independent ambulation by the middle of the patient’s 2nd decade. This condition also results in fatal cardiac or respiratory failure by the 3rd decade [2]. DMD has been well characterized as a disease that primarily affects the development of skeletal muscle in the affected patients; however, this disease also has a significant impact on osseous tissue. The pathophysiology responsible for deficiencies in bone tissue seen in these patients has not been extensively studied despite the detrimental effect that it can have on patient outcomes. Studies have demonstrated that DMD patients have a significantly higher risk of long bone fracture compared to age-matched controls with the prevalence ranging from 20–45% [3,4,5]. Similar results were also found in DMD patients without steroid therapy [3,4,6]. Furthermore, bone quality appears to be affected in these DMD patients prior to a decrease in ambulatory status [3], which suggests that decreased bone mechanical stimulation secondary to diminishing ambulation may not be the sole cause of the change in bone structure. Therefore, it is speculated that diseased muscle itself, e.g., as a result of muscle wasting, could contribute to the pathogenesis of bone tissue deterioration in DMD.

The mdx mouse (dystrophin−/−), a common DMD murine model [7,8], has a complete loss of dystrophin but has utrophin compensation and does not recapitulate the severe phenotype of DMD patients, especially in mdx mice less than one year of age. The dKO (dystrophin/Utrophin double knockout; dystrophin−/−, utrophin−/−) mouse model is a more severe mouse model of DMD than the mdx mouse [9,10,11,12,13,14]. These mice present with numerous clinically-relevant manifestations, including muscle wasting, crippling, kyphosis, heart failure, and other life-threatening phenotypes, similar to DMD patients [9,10]. More importantly, we have reported that dKO mice exhibit a spectrum of degenerative changes outside skeletal muscle including bone, articular cartilage, and intervertebral discs [15]. We have also reported that dKO mice have a reduced capacity for bone healing and exhibit spontaneous heterotopic ossification in their hind limb muscles [15,16], supporting our contention that the dKO mouse represents a better animal model to study muscle and bone comorbidities of DMD. It remains unclear whether the defect in bone homeostasis and in bone healing, observed in DMD patients and in dKO mice, can be attributed to an intrinsic bone turnover problem or whether it is a secondary effect due to muscle loss (sarcopenia) present in this animal model. Our recent study has shown that the bone problems occur in this animal model at a later time point than the muscle histopathologies suggesting these bone abnormalities are likely secondary to the muscle pathologies [17]. We hypothesize that systemic blood-borne factor(s) due to muscle pathology accumulated in dystrophic mice play an important role in mediating the bone deficiency of DMD. 

In this study, we investigated whether the bone abnormalities observed in dKO mice can be rescued through parabiotic pairing which will rejuvenate the dKO microenvironment by creating a shared circulation between dKO and young WT and mdx animals. Parabiosis is a process in which two mice are surgically connected to establish a shared blood circulation thus allowing for the exchange between the two animals of circulating blood, immune factors, circulating stem cells, hormones, growth factors and a myriad of other circulating molecules [18,19,20]. This surgical tool has also been employed by investigators to study the effect of aging on skeletal muscle stem cells by examining the effect that circulating factors from a young mouse have on a conjoined older animal [21,22,23]. Our results indicate that heterochronic parabiosis with young mdx mice significantly increased bone mass and improved bone micro-structure in old dKO-hetero mice. Furthermore, heterochronic parabiosis with WT C56/10J mice significantly improved tibia bone defect healing in dKO-homo mice.

## 2. Results

### 2.1. Cross-Circulation in Parabiotic Pairs

Two heterochronic pairs and two young mdx isochronic pairs died at two weeks after surgery due to “parabiotic intoxication” [24] which include the following features (pale, anemic, and shriveled appearance of one parabiont and swollen, plethoric appearance of the other parabiont); and one homo isochronic pair was sacrificed due to severe skin infection. A total of eight pairs of heterochronic parabionts of old dKO-hetero (12–14 months) and young mdx (3–5 months) mice, eight pairs of isochronic control parabionts of young/young mdx (eight pairs), six pairs of isochronic control parabionts of old/old dKO-hetero mice, and six pairs of heterochronic parabionts of four weeks old dKO-homo mice with C57BL/10J WT mice survived till the end of the experiment.

**Figure 1:**
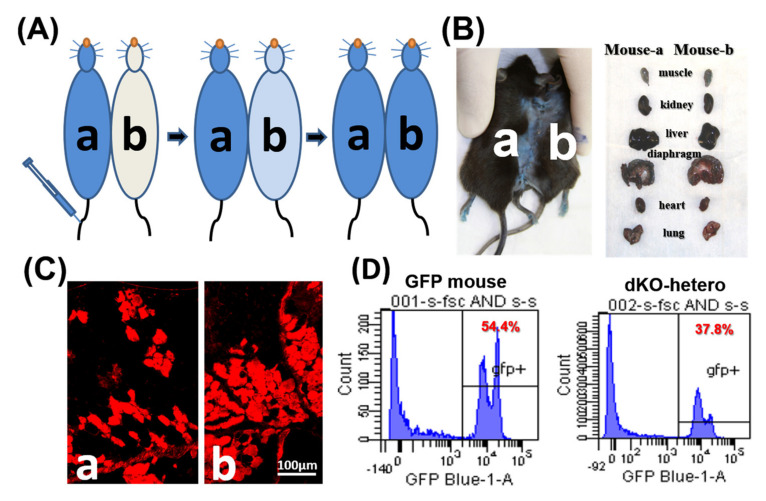
The establishment of parabiotic models. (**A**) Mdx (a) and dKO-hetero mice (b) were joined surgically via parabiosis. Two weeks after surgery, Evans blue (350 μL) were injected into the tail vein of mdx mice (a). (**B**) 20 h after injection, both mice showed blue stain on their skin and internal organs. (**C**): Gastrocnemius muscles were sectioned and fluorescence signal (red) of Evans blue that leaked into the damaged myo-fibers were detected in both dystrophic mice. (**D**): WT-GFP mice were joined with dKO-hetero mice surgically via parabiosis for 2 weeks. Peripheral blood was collected and depleted with the red blood cells. GFP signals were tested via FACS. Mixed GFP (−) and GFP (+) cells were detected in the peripheral blood of each partner in dKO-hetero/GFP mice pairing demonstrating shared circulating between paired mice.

To confirm the formation of a shared blood circulation between the parabiotic animals, one pair of control parabionts of old/old dKO-hetero mice were tested at two weeks after parabiosis. Cross-circulation (chimerism) in paired animals was confirmed by tracking the flow of EBD from mouse “a” to mouse “b” following intravenous (tail vein) injection (Figure 1A). After the injection of EBD, mouse “a” skin turned immediately blue, whereas the un-injected counterpart remained normal in colored at the five-min time point. 20 h after the injection, both mice demonstrated blue staining of their skin and internal organs (Figure 1B). As both mice are dystrophic, gastrocnemius muscles were sectioned and fluorescence signal (red) of EBD that leaked into the damaged myo-fibers was detected in muscle fibers from both mice (Figure 1C). To further validate peripheral blood chimerism, a dKO-hetero mouse was paired with a GFP transgenic mouse (C57BL/6-Tg [CAG-EGFP] 10sb/J, Jackson Laboratory). This transgenic mouse line has widespread EGFP fluorescence, with the exception of erythrocytes and hair. The traffic of the GFP positive nucleated blood cells between these paired mice were analyzed by the presence of GFP positive cells in red blood cell depleted peripheral blood from both animals via fluorescence activated cell sorting (FACS) at two weeks after parabiosis procedure. We found around 40% GFP-positive cells in the peripheral blood of paired dKO-hetero mouse. Similarly, around half of the blood cells in paired GFP mouse are GFP-negative, which clearly indicate the exchange of blood cells between these paired animals (Figure 1D). Taken together, these data demonstrated that a successful cross-circulation can be established between the paired mice two weeks after parabiosis.

### 2.2. Increased Bone Mass and Improved Bone Micro-Structure in Old dKO-Hetero Mice after Pairing with Young Mdx Mice

**Figure 2:**
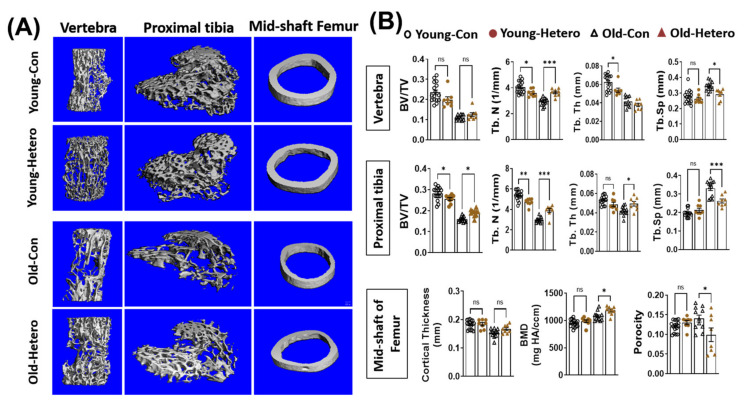
Heterochronic parabiosis reduces bone loss in old dKO-hetero mice. (**A**): Representative 3-D reconstructed images of the L6 vertebra, epiphyseal part of tibia, and mid-shaft of femur. (**B**) Quantification of bone microarchitectural parameters. Young-Con: young mdx mice in young mdx/young mdx parings; Young-Hetero: young mdx mice in old dKO-hetero/young mdx parings; Old-Con: old dKO-hetero in old dKO-hetero/old dKO-hetero parings; Old-Hetero: old dKO-hetero in old dKO-hetero/young mdx parings. BV/TV: bone volume/tissue volume; Tb. N: trabecular number; Tb. Th: trabecular thickness; Tb. Sp: trabecular spacing. Data were expressed as Mean ± SEM; * *p* < 0.05, ** *p* < 0.01, *** *p* < 0.001.

We have reported the reduction of bone tissue, in both the lumber vertebra and the epiphysis of the proximal tibia, in dKO mice when compared with age-matched mdx and WT controls [15]. In this study, we observed significantly reduced bone fraction (BV/TV), trabecular number (Tb. N), trabecular thickness (Tb. Th), and higher trabecular spacing (Tb. Sp) in both the lumber vertebra and proximal tibia in old-dKO-hetero mice (old-con) when compared with young-mdx mice (Young-con) (Figure 2). Interestingly, although it did not reach significance, lower bone mineral density (BMD) (Figure 2). These data demonstrated deleterious bone quality in dKO-hetero mice during the disease progression. Examination of the old dKO-hetero mice in old/young pairings (Old-Hetero) revealed an increase in bone mass and improved bone micro-structure in the lumber vertebra, epiphysis of the proximal tibia, and mid-shaft of femur when compared with the old dKO mice in old/old pairings (Old-con, Figure 2A). Quantitative analyses confirmed that old dKO mice in old/young pairings had: (1) significantly more Tb. N and less Tb. Sp in their lumber vertebra when compared with old dKO mice in old/old pairings (Figure 2B, Vertebra); (2) more BV/TV, Tb. N and Tb. Th, and less Tb. Sp in their epiphysis of the proximal tibia when compared with old dKO mice in old/old pairings (Figure 2B, Proximal tibia); and (3) significantly higher BMD and reduced porosity in the cortical bone of mid-shaft of femur when compared with old dKO mice in old/old pairings (Figure 2B, Mid-shaft of femur). However, no significant differences were observed in BV/TV and Tb. Th in lumber vertebra, and cortical thickness in mid-shaft of femur between groups. Meanwhile, we observed decreased bone mass and deteriorated bone micro-structure in young mdx mice in old/young pairings (Young-Hetero) when compared with the young mdx mice in young/young parings (Young-Con, Figure 2B). Quantitative analyses showed less Tb. N and Tb. Th in lumber vertebra; lower BV/TV and less Tb. N in epiphysis of the proximal tibia in young mdx mice that were paired with old dKO-heteros when compared with young mdx controls. Taken together, these data demonstrated that the pairing of old dKO hetero mice with young mdx mice significantly reduced bone loss in trabecular bone in the old dKO hetero mice and simultaneously causing deleterious bone changes in young mdx mice, although in a lesser extent. 

### 2.3. Improved Bone Healing of dKO-Homo Mice after Paring with WT Mice

**Figure 3:**
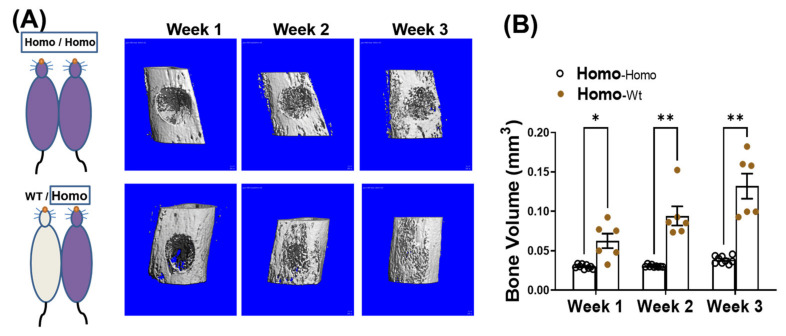
Pairing with WT mice improves bone healing in dKO-homo mice. (**A**): Representative 3-D reconstructed images of bone defects in proximal tibia. First row, bone defect areas of dKO-homo mice in homo/homo isochronic control pairings; second row, bone defect areas of dKO-homo mice in homo/WT heterochronic parings. (**B**) Quantification of bony-in-growth within defect areas. Data were expressed as Mean ± SEM; * *p* < 0.05, ** *p* < 0.01.

We have reported that in addition to progressive bone loss, dKO-homo mice also demonstrated inferior bone healing capability [15]. To test if systemic circulating factors accumulated in DMD also play a role in causing the inferior bone healing capability, four weeks old dKO-homo mice were paired with age matched WT mice, and a spherical, unicortical defect was surgically created in the proximal tibia of the dKO mice two weeks after parabiosis. The mice were followed with serial micro-CT scans to monitor bony in-growth at one, two, and three weeks post-operatively. The dKO-homo mice in homo/WT pairings display a significantly higher bony-in-growth started from week 1, and through all time points compared to the dKO homo mice in homo/homo isochronic control pairings (Figure 3A). At week three, very few bony-in-growth were observed in isochronic homo/homo pairs, while almost complete healing was observed in homo mice paired with WT mice (Figure 3B). These data demonstrated that the reduced bone healing in the proximal tibia observed in dKO-homo mice is improved through parabiosis with a WT animal.

### 2.4. Quantification of Bone Marrow Mesenchymal Stem Cells (CFU-F) and Hematopoietic Stem Cells (CFU-GM) Isolated from the Bone Marrow

**Figure 4:**
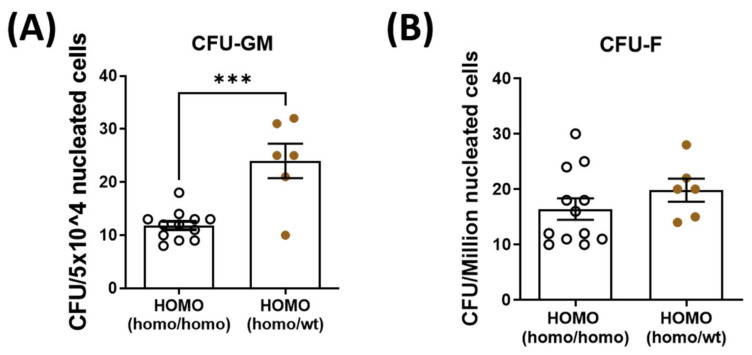
Quantification of bone marrow hematopoietic stem cells (CFU-GM) and mesenchymal stem cells (CFU-F) isolated from the bone marrow. Bone marrow hematopoietic stem cells and mesenchymal stem cells were isolated and quantified from the contralateral tibia. (**A**): Hematopoietic stem cell quantity was significantly increased in the dKO-homo mice after paired with WT mice. (**B**): No significant difference was noted in the quantity of bone marrow-derived mesenchymal stem cells between the two groups. Data were expressed as Mean ± SEM; *** *p* < 0.001.

To decipher the role of parabiosis in bone healing, bone marrow mesenchymal stem cells and hematopoietic stem cells were isolated and quantified from the contralateral tibia of dKO mice. Hematopoietic stem cell quantity was significantly increased in the dKO-homo mice after pairing with WT mice (Figure 4A). There was no significant difference in the quantity of bone marrow-derived mesenchymal stem cells between the two groups (Figure 4B).

## 3. Discussion

Most of the work toward developing a treatment for DMD over the last 20 years has focused on skeletal muscle repair, with little effort being directed toward understanding the other musculoskeletal abnormalities observed in DMD patients. Despite the assumption that bone abnormalities are a secondary consequence to the muscle pathology, it is important to note that most DMD patients become wheelchair-bound after they experience a bone fracture, which demonstrates the importance of increasing our understanding of these bone abnormalities in order to improve the quality of life of DMD patients. 

Parabiosis is a process in which two mice are surgically connected to establish a shared blood circulation thus allowing for the exchange between the two animals of circulating blood, immune factors, circulating stem cells, hormones, growth factors and a myriad of other circulating molecules. Parabiosis has been used to evaluate disease progression in knockout mice by conjoining a healthy WT mouse to a diseased mouse model [18,19,20]. This surgical tool has also been employed by investigators to study the effect of aging on skeletal muscle stem cells by examining the effect that circulating factors from a young mouse have on a conjoined older animal [21,22,23]. We posit that accumulation of some substances in the circulation during the disease progression in DMD play a role in the progression of DMD phenotypes.

Heterochronic parabiosis studies were performed based on dydy dystrophic mouse models at early 60s with controversial results. Hall et al. [25] reported significantly prolonger life expectancy from eight to ten weeks to 24 weeks in males and 25 weeks in females. However, Pope et al. reported the opposite [26,27]. Although, Pope et al. did not observed prolonged life span after parabiosis with WT animals, nor any noticeable physical improvement, they observed significantly less severe muscular dystrophic lesions [28]. Although controversial, these early studies indicate that some blood-borne factors may be related to the progression of muscle lesions of muscular dystrophy. Unfortunately, no changes of their bone phenotypes were reported and no further follow-up studies published thereafter.

In the current study, parabiosis were used to answer the questions that if blood-borne factors may also be related to the development/progression of the bone deficiencies observed in dystrophic mice. Surprisingly, we observed significantly improved bone quality in old dKO-hetero mice that paired with young mdx mice and improved bone healing property in dKO-homo mice that paired with WT mice, which strongly suggests that blood borne factors are responsible, or at least play a role, for the bone abnormalities in DMD. Moreover, we also observed deleterious bone changes in young mdx mice that were paired with old dKO-hetero mice. These observations further indicate that it is indeed the circulating factors in the older dKO animals that are causing the deleterious bone changes. The next logical question to ask is which circulating factors are responsible to alleviate the bone abnormalities in old-dKO mice after heterochronic parabiosis procedure. To address that, the generalized “omics” approaches to reveal such factor(s) or cell(s) is needed based on this heterochronic parabiosis model. Although out of the scope of the current study, several studies have already shad lights on this topic. One study found that IL-6, a pro-inflammatory cytokine, significantly increased in the circulation of mdx mice and in DMD patients [29]. Blockage of circulating IL-6 via a neutralization Ab normalized the bone resorption parameters in ex-vivo cultured calvarial bones from mdx mice [29]. Elevated circulating IL-6 maybe one of those bone-deteriorating factors. In addition to highly expressed in bone, recent studies have shown higher expression levels of RANK and RANKL from dystrophic skeletal muscles [30,31,32]. Systemic usage of anti-RANKL antibody or OPG, which is a decoy receptor for the RANKL, significantly mitigated skeletal muscle pathologies along with improvement of bone stiffness [31]. Although it is still not known if circulating levels of RANKL and/or OPG are elevated in DMD, and weather muscle-derived RANKL directly affects bone, these studies have emphasized the mutual cohesion and common signaling pathways between bone and skeletal muscle in DMD. Elucidating the muscle/bone cross talk is of great importance to understand the muscle and bone comorbidities in DMD. Most recently, we have reported significantly different expressed/secreted bone regulating myokines from dystrophic skeletal muscles in DMD mouse models compared to WT controls, which includes FGF21, LIF, IL-6, Myostatin, and RANKL etc. [33]. We have further shown that when FGF21 was neutralized using an anti-FGF21 antibody, progressive bone loss in both weight-bearing and non-weight bearing parts were significantly reduced in dystrophic mice [34]. Taken together, our current findings along with others have suggested that circulating factors accumulated in DMD may play a role in deteriorating bone quality. Further investigations are needed to identify and characterize those factors and elucidate their cellular and molecular mechanisms that affect bone. We have nevertheless provided evidences that blood-borne factor(s) accumulated in dystrophic mice play an important role in mediating the bone deficiency of DMD. This is the first step in linking circulating factors or cells with the bone abnormalities in DMD. After that, the generalized “omics” approaches to reveal such factor(s) or cell(s) is needed based on this heterochronic parabiosis model.

In addition to the circulating factors, it is possible that circulating cells from young mdx mice and/or WT mice might also contribute to the improved bone quality and bone healing in paired dKO mice. However, under current experimental settings, we cannot investigate the contribution of circulating cells due to lack of tracing markers. Further investigations by pairing dKO mice with reporter mouse that has specific genetic markers, will help to address those questions. Those investigations should be aimed to answer the questions that: (1) if there are circulating cells actually participate in the bone turnover and/or new bone formation in bone defect repair; (2) What is the characteristics of those circulating cells? Are those cells circulating progenitor cells that could actually integrate into the newly formed bone tissues or circulating immune cells that transitionally facilitate bone turn-over and bone repair processes? (3) If there are circulating cells that not directly participate the bone turn-over and bone repair processes but integrated into other organs which indirectly affect bone turn-over and bone repair via modifying the systemic milieu. In terms of circulating osteogenic progenitor cells, based on the published data [35,36], even there are some, they are with a very low frequency. Although not designed to address the pathogenesis of bone abnormalities in DMD, several parabiosis studies by using specific reporter mice have been conducted with the attempt to answer the question that if there are circulating osteogenic progenitor cells that participate to bone turn-over and bone healing. Kumagai et al. [37] paired wild-type female mice with GFP transgenic male mice to test the share of GFP (+) cells during bone fracture healing. Although in a low percentage and the nature of the infiltrating GFP (+) cells are still not clear, they clearly observed GFP (+) cells not only in normal non-fractured bone but also in fractured bone. However, Ivana et al. [20] did not observed circulating osteoprogenitor cells that were shared in parabiosis pairs. Interestingly, both studies [20,37] observed GFP (+) cell integration in the bone marrow of the paired partner and these GFP (+) cells were of hematopoietic origin, more specifically belonging to the osteoclast lineage. In the current study, we also observed significantly increased hematopoietic stem cells but not mesenchymal stem cells in the bone marrow of dKO mice after parabiosis, which suggests the possibility that parabiosis may affect the impaired bone healing process in dKO mice via modulating osteoclastogenesis or osteoclast functions. Further investigation is needed to clarify this possibility. Moreover, it has been demonstrated that the dystrophic mice are chronically hypoxia and hypercapnic and it has a significant higher hematocrit as a compensatory mechanism [38]. The increased hematopoietic stem cells in bone marrow may also give rise to specific posterior linages such as red blood cells, which may contribute to the general improved parameters observed. More detailed dissection of the changes of different hematopoietic linages is needed. 

Another explanation of the improvements seen in parabiosed old dKO-hetero and dKO-homo mice might due to the improved nutrition provided by the relatively healthier or normal partner. At the moment, we could not exclude this possibility due to lack of a reliable system to monitor and control the food consumption of the two paired animals. However, this explanation seems improbable with the observation that the stronger parabionts always dominant the movement in the cage and the weaker part of the parabionts may not take as much food as it normally does, which suggests that the weak dystrophic mice paired with WT mice may theoretically take less food than their isochronic controls. Moreover, no significant body weight changes were found among groups, which also suggest that the nutritional explanation is less likely.

There are some limitations of the current study. First, functional assays, such as bone mechanical tests both on cortical and cancellous bones, will give us more information regarding the changes of bone mechanical properties in addition to the 3D morphometric changes after parabiosis. Second, the overall health and/or skeletal muscle functionalities may also contribute to the general improvement of the bone parameters that we observed. More detailed systemic parameters such as body weight, food consumption, motor function and specific muscle contractility before and after parabiosis will help to refine the interpretation of the data. Third, one may argue that the beneficial effects seen in older dKO mice is just a reflection of the younger mice on older mice and it is not specific to dystrophic mice. A series rigorous age-matched pairing maybe helpful to rule out this possibility. Even that, it is also of great interests to identify those factors that represents the differences between young and old. Fourth, although data presented in the current study strongly suggested that the blood-borne factors or progenitors may play an important role in causing the low bone quality and defective bone healing of DMD, the exact mechanism(s) is still unclear. Further investigations based on this model are needed to elucidate: (1) whether absence or accumulation of circulating soluble mediator(s) in dystrophy negatively affects bone homeostasis; (2) whether defective circulating cellular mediator(s), such as inflammatory cells and/or progenitor cells may play a role in regulating bone turnover and bone healing process; or (3) a combination of both. Finally, high-throughput -omics based analytic assays on blood, skeletal muscle and bone tissues should be performed to identify possible factor(s) that contribute to the bone deterioration response induced by the lack of dystrophin.

Nevertheless, the current study demonstrated that the parabiosis model provides a useful, and potentially powerful, model for investigating further the treatment options targeting blood-borne factors in managing musculoskeletal deficiencies in DMD. The findings of a robust effect on bone quality and bone healing in dystrophic mice after parabiosis raises the possibility that strategies could be devised to experimentally or therapeutically manipulate circulating factors in a manner that regulating bone homeostasis and even promotes regeneration.

## 4. Materials and Methods

### 4.1. Animals

The University of Pittsburgh’s Institutional Animal Care and Use Committee (IACUC, University of Pittsburgh, Pittsburgh, PA, USA) approved all experiments. Wild-type (C57BL/10J) mice and GFP transgenic mice (C57BL/6-Tg [CAG-EGFP] 10sb/J) were obtained from the Jackson Laboratory (Bar Harbor, ME, USA). Three different animal models of DMD were utilized in this study: mdx (dystrophin−/−) mice have mild skeletal muscle defects and potent regenerative capacity, whereas dKO-homo (dystrophin−/−, utrophin−/−, dystrophin and utrophin double knock out) mice display severe musculoskeletal changes but a very limited lifespan (8 to 10 weeks in general), while dKO-hetero (dystrophin−/−, utrophin+/−) mice display a similar phenotype as dKO-homo with a longer life expectancy. All dystrophic animals were derived from our in-house colony originally from Dr. Davies [39]. The methods were performed in accordance with the approved guidelines and regulations. Animals of both genders were used for this study, and only mice with the same gender were paired by parabiosis.

To test if systemic circulating factors accumulated in DMD play a role in the progressive bone loss, three types of parabiosis pairs were performed, designed as follows: (1) heterochronic parabionts of old dKO-hetero (12–14 months) and young mdx (3–5 months) mice (10 pairs); (2) isochronic control parabionts of young/young mdx (10 pairs); as well as (3) isochronic control parabionts of old/old dKO-hetero mice (7 pairs). Three months after pairing, both animals were sacrificed, and bone tissues were collected for radiographical and histological analyses.

To test if parabiosis can affect the bone healing, six pairs of heterochronic parabionts of four weeks old dKO-homo mice with same aged C57BL/10J WT mice were performed. The bone defects on proximal tibia were created on both mice two weeks after parabiosis. The bone healing process was monitored weekly by in vivo micro-CT scanning for three weeks. The animals were sacrificed at nine weeks of age and bone tissues were collected for further histological analysis. Two groups of isochronic parabionts, WT/WT and dKO-homo/dKO-homo, four pairs each, served as controls.

### 4.2. Parabiosis Procedure and Chimera Generation

Parabiosis surgery was performed as described previously [23,40] with some modifications. Briefly, the animals were anesthetized by inhalation of oxygenated isoflurane (2% isoflurane with 1.5% O_2_ L/min). Following the required surgical preparations and shaving of the skin along the flanks of mice to be joined, matching skin incisions from the olecranon to the knee were made along the opposing flanks of each mouse. The subcutaneous tissue was dissected to create a 0.5 cm free skin flap. The limbs of the two animals were joined laterally by silk sutures, followed by skin closure using continuous nylon sutures. Parabiotic pairs were housed separately and fed with mushed standard rodent chow with adequate water. Diet and water were offered ad libitum in a low-profile dish on the cage floor to make sure both parabionts can equally get nutrition. To confirm the formation of a shared blood circulation between the parabiotic animals (stable chimerization), at 14 days post-surgery, Evans blue dye (EBD), which binds to serum albumin were injected into the tail vein of one paired mouse and the successful transfer of the EBD into the other paired mouse were assayed in the circulation and tissues. The EBD transfer to the paired animal was visualized by the EBD dye on the skin and internal organs and the florescence signal from the damaged dystrophic skeletal muscle (Gastrocnemius-GC) [41]. Furthermore, to further confirm peripheral blood chimerism, a dKO-hetero mouse was paired with a GFP transgenic mouse, and the transfer of GFP positive cells were analyzed by the presence of GFP positive cells in red blood cell depleted peripheral blood from both animals via fluorescence activated cell sorting (FACS).

### 4.3. Tibia Bone Defect Healing

Two weeks after parabiosis, mice were sedated as above, and unicortical, spherical defects (non-critical sized) were created using a 0.9 mm burr (Fine Science Tools Inc, Foster City, CA, USA) on the proximal tibia of the lateral knees of the paired mice, as described previously [15]. Bone in-growth within the defect area was evaluated with serial micro-CT on postoperative days of 7, 14 and 21 days.

### 4.4. Micro-CT Bone Histomorphometric Assessment

Lumbar spine, femur, and tibia were collected at sacrifice and fixed in 4% paraformaldehyde for 24 h. Micro-CT scans were performed to evaluate bone volume (BV) and architectures of lumbar vertebrae, mid-shaft of femur, metaphyseal part of proximal tibia using Viva-CT 40 (SCANCO Medical, Wangen-Brüttisellen, Switzerland) with settings: energy 70 kV, intensity 114 µA, integration time 300 ms, and isotropic voxel size of 10.5 µm as descripted previously [15,33]. Region of interests (ROI) including the trabecular bones from lumbar vertebrae and metaphyseal part of proximal tibia, and cortical bones from mid-shaft of femur. Multiple bone microarchitectural parameters of different bones were determined. Bone fraction (bone volume/tissue volume, BV/TV), trabecula number (Tb. N), trabecular thickness (Tb. Th), and bone mineral density (BMD) were analyzed for trabecular bones; cortical bone thickness, bone area, porosity, and BMD were analyzed for cortical bones. For the evaluation of bone in-growth in tibia defects, a cylinder ROI was created (0.9 mm diameter, 0.6 mm depth), and the total bone volume within the bone defect area was calculated by measuring the mineralized bone tissue as descripted previously [42]. 

### 4.5. Colony Forming Unit (CFU) Assay

To evaluate progenitor cells, both mesenchymal stem cells (MSCs) and the hematopoietic stem cells (HSCs) from bone marrow (BM) were quantified by the colony-forming unit-fibroblast (CFU-F) and CFU-granulocytic-monocyte (CFU-GM) assays respectively. Briefly, single-cell suspension of BM cells from femur and tibia was collected. 10^5^ nucleated BM cells per animal were seeded in triplicate into a 6 well-plate with 1.1 mL of MethoCult M3534 methylcellulose medium (StemCell Technologies, Vancouver, BC, Canada) or with Complete MesenCult medium (mouse, StemCell Technologies). MethoCult M3534 and complete MesenCult media have been formulated to support the optimal growth of granulocyte/macrophage precursor cells and mesenchymal stem cells respectively. After seven to ten days in culture, the cell colonies in MethoCult medium (CFU-GM) were imaged using stereo microscope (Olympus SZX16, Olympus, Tokyo, Japan), and colonies (>30 cells) were counted. The cell colonies in MesenCult medium (CFU-F) were stained with Giemsa stain (Sigma-Aldrich, St. Louis, MO, USA) and counted. Colony recognition and enumeration were performed according to StemCell Technologies guidelines.

### 4.6. Statistical Analysis

Data are expressed as Mean ± SEM. GraphPad Prism 7 software was used for statistical analysis. Comparison between two groups was assessed by non-parametric Mann-Whitney U-test. Comparison among ≥3 groups was assessed by one-way ANOVA with Bonferroni Post Hoc test. *p* < 0.05 was considered significant. 

## 5. Conclusions

In conclusion, heterochronic parabiosis with young mdx mice significantly increased bone mass and improved bone micro-structure in old dKO-hetero mice, which showed progressive bone deterioration. Furthermore, heterochronic parabiosis with WT C56/10J mice significantly improved tibia bone defect healing in dKO-homo mice. These results suggest that systemic blood-borne factor(s) and/or progenitors from WT and young mdx mice can influence the bone deficiencies in dKO mice. These results also suggest that understanding these circulating factors or progenitor cells that are responsible to alleviate the bone abnormalities in dKO mice after heterochronic parabiosis, might be useful for the management of poor bone health in DMD.

## Data Availability

The original data can be obtained on a request from the corresponding authors.
